# The Effect of Constraint-Induced Movement Therapy Combined With Repetitive Transcranial Magnetic Stimulation on Hand Function in Preschool Children With Unilateral Cerebral Palsy: A Randomized Controlled Preliminary Study

**DOI:** 10.3389/fnbeh.2022.876567

**Published:** 2022-04-05

**Authors:** Qianwen Wu, Tingting Peng, Liru Liu, Peishan Zeng, Yunxian Xu, Xubo Yang, Yiting Zhao, Chaoqiong Fu, Shiya Huang, Yuan Huang, Hongyu Zhou, Yun Liu, Hongmei Tang, Lu He, Kaishou Xu

**Affiliations:** ^1^Department of Rehabilitation, Guangzhou Women and Children's Medical Center, Guangzhou Medical University, Guangzhou, China; ^2^School of Medicine, South China University of Technology, Guangzhou, China; ^3^Department of Rehabilitation, Kunming Children's Hospital, Kunming, China

**Keywords:** constraint-induced movement therapy, repetitive transcranial magnetic stimulation, preschool children, unilateral cerebral palsy, hand function

## Abstract

Constraint-induced movement therapy (CIMT) combined with repetitive transcranial magnetic stimulation (rTMS) have shown great potential in improving function in schoolchildren with unilateral cerebral palsy attributed to perinatal stroke. However, the prospect of application in preschool children with unilateral cerebral palsy (UCP) attributed to various brain disorders remains unclear. In this prospective, assessor-blinded, randomized controlled study, 40 preschool children with UCP (aged 2.5–6 years) were randomized to receive 10 days of CIMT combined with active or sham rTMS. Assessments were performed at baseline, 2 weeks, and 6 months post-intervention to investigate upper limb extremity, social life ability, and perceived changes by parents and motor-evoked potentials. Overall, 35 participants completed the trial. The CIMT plus active stimulation group had greater gains in the affected hand function (range of motion, accuracy, and fluency) than the CIMT plus sham stimulation group (*P* < 0.05), but there was no significant difference in muscular tone, social life ability, and perceived changes by parents between the two groups (*P* > 0.05). In addition, there was no significant difference in hand function between children with and without motor-evoked potential (*P* > 0.05). No participants reported severe adverse events during the study session. In short, the treatment of CIMT combined with rTMS is safe and feasible for preschool children with UCP attributed to various brain disorders. Randomized controlled studies with large samples and long-term effects are warranted.

## Introduction

Cerebral palsy is the most common physical disability in childhood, occurring in 2.5–3.5 per 1,000 live births and with complicated etiology (Bax et al., 2005; Li et al., 2021). Unilateral cerebral palsy (UCP), which mainly affects the function of children's lateral extremity, accounts for 44% of the cases (Stavsky et al., 2017). The main manifestations in children are motor impairments and may encompass heterogeneous clinical performance including impairment of communication, cognition, or sensation (especially tactile sensation), the difficulty of daily task performance, and quality of life (Senst, 2014).

Over the last decade, studies on interventions for children with UCP have grown exponentially. It was indicated that the effect of most treatments for upper limb function in hemiplegic patients is induced by the principles of task- and context-specific motor learning and repetition (Veerbeek et al., 2014). One of the most popular treatments among clinicians and researchers is constraint-induced movement therapy (CIMT), with an emphasis on constraining the unaffected extremity and coupling task-related practice with the affected upper extremity, and increasing evidence proved the effect of CIMT in children with UCP (Reedman et al., 2017; Hoare et al., 2019; Ilieva and Ilieva, 2020; Simon-Martinez et al., 2020). What is more, it was proved that CIMT might promote neural remodeling and thereby improve motor function (Liu et al., 2021). However, the findings on the effect of CIMT on improving bimanual coordination are controversial (Reid et al., 2015; Hoare et al., 2019). In addition, it has not been proven to have much effect on improving decreasing muscle tone (Reid et al., 2015). Studies have shown that neuromodulation technology such as repetitive transcranial magnetic stimulation (rTMS), which acts directly on the central nervous system, may yield a great impact on the overall motor ability and decrease the muscle tone of children with cerebral palsy (Boddington and Reynolds, 2017; Gupta and Bhatia, 2018; Parvin et al., 2018; Rajak et al., 2019).

Children with UCP demonstrate atypical patterns of corticospinal tract development and organization, which leads to an imbalance in excitability between the affected and unaffected hemispheres (Berweck et al., 2008; Chen et al., 2016). These neural changes may underlie the limitations in upper extremity function and social life ability (Holmström et al., 2010). Given that rTMS depolarizes neurons by means of strong, short magnetic pulses, aiming to suppress or facilitate cortical excitability depending on electrode polarity, it may make up the shortcomings of CIMT (Klomjai et al., 2015; Lefaucheur et al., 2020). Indeed, the effect of CIMT combined with rTMS has been proven in improving behavioral function and neurophysiologic responses in school-aged children with UCP attributed to perinatal strokes (Kirton et al., 2016).

Furthermore, emerging evidence suggested that the CIMT was more effective during the early developmental period (Reid et al., 2015; Boddington and Reynolds, 2017). To our best of knowledge, there are few studies to evaluate the effect of CIMT combined with rTMS on the treatment response in young children (Novak et al., 2020). Due to the immature pattern of hand function and poor self-control, preschool children with UCP, who are often affected by joint reaction and mirror movements, i.e., involuntary imitations of unilateral voluntary movements, can easily be affected by the motor pattern of the affected side. This period may be critical for more effective rehabilitation. On the other hand, most studies focused on perinatal strokes, although UCP has complicated pathogenic factors.

To fill this gap, we carried out a randomized controlled study to evaluate the effect of CIMT combined with rTMS in preschool children with UCP attributed to various brain disorders.

## Methods

The design of this study was a prospective, assessor-blind, and randomized controlled trial, which was registered at chictr.org (ChiCTR1900021924). The institutional research ethics board approval was obtained from Guangzhou Women and Children's medical center, and written informed consent was obtained from the legal representative of each participant before enrollment.

### Participants

**Figure 1:**
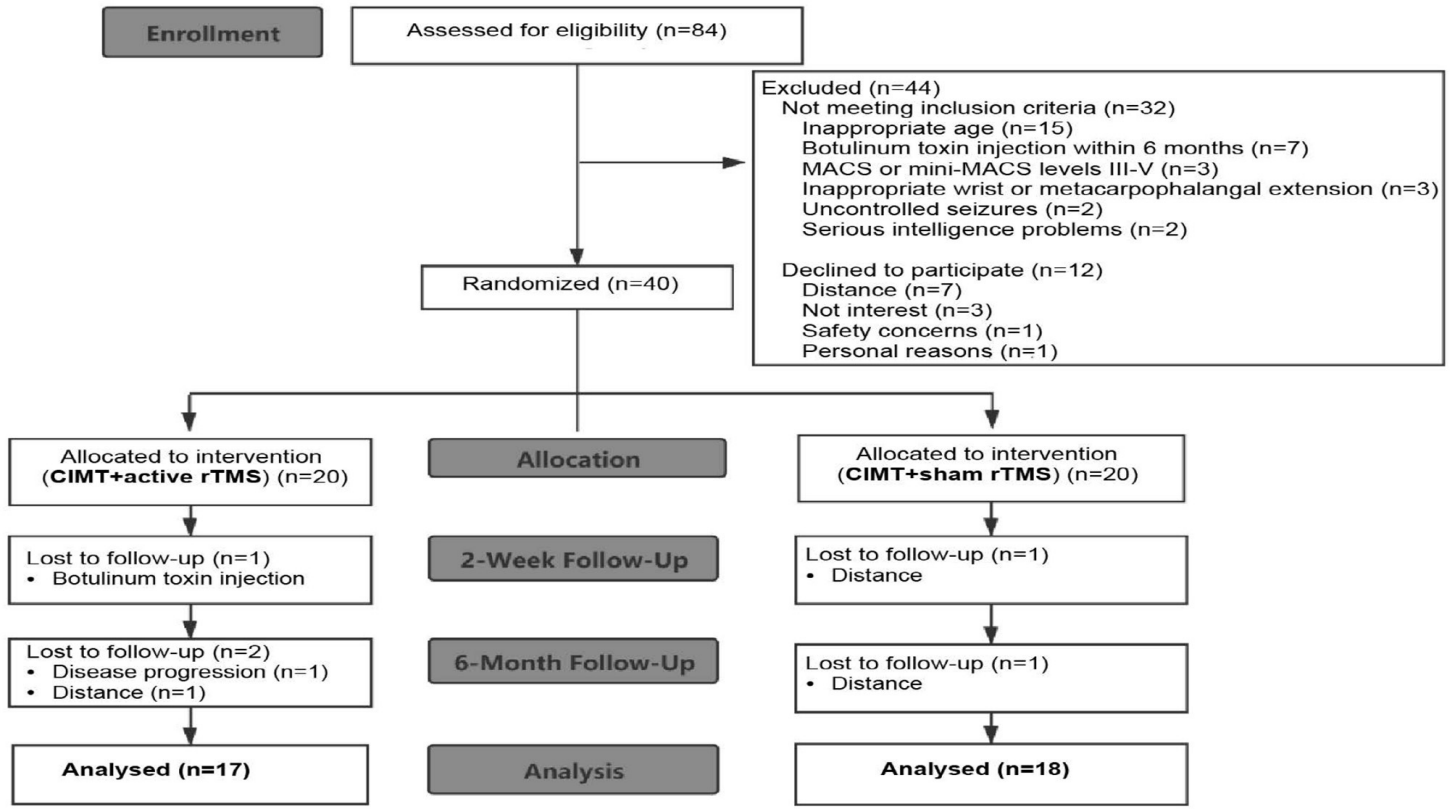
Study flow diagram.

Eighty-four preschool children with UCP were recruited through the goal-directed, peer-supported CIMT camp program. Recruitment occurred from March 25, 2019 to August 31, 2019. Inclusion criteria were as follows: (i) aged 2.5–6 years; (ii) Manual Ability Classification System levels I-II or Mini-Manual Ability Classification System levels I-II; (iii) ≥20° wrist active extension and ≥10° metacarpophalangeal active extension from full flexion; (iv) a 20–80% difference of global rating scale scores between the affected and unaffected hands; and (v) written informed consent. Participants were excluded if they met any of these criteria: (i) other neurological diagnosis; (ii) uncontrolled seizures; (iii) severe sensory impairment or visual problems; (iv) contraindication for rTMS (Wassermann, 1998; Kirton et al., 2008); (v) upper limb surgery; or (vi) botulinum toxin treatment within 6 months. A total of 40 children met the inclusion criteria. Thirty-five children completed the study in the end (with 17 children in the CIMT plus active stimulation group). The flow of patients is summarized in Figure 1.

### Design

Participants were randomly assigned to CIMT plus active or sham stimulation groups (1:1) in an unbiased manner using a random number table produced by Statistical Product and Service Solutions for Windows (release 25.0, SPSS). Assessments and administration of the functional scales and questionnaires were performed by two independent assessors who had received training and certification of the study measures. CIMT assignment was concealed from the assessors. Assessors were blind to rTMS assignment. Endpoints were assessed at the baseline visit, 2 weeks, and 6 months postintervention.

### Interventions

All the involved children participated in the 10 consecutive days of goal-directed CIMT camp, active or sham rTMS was applied independently in a separate room before daily CIMT therapy. During the rTMS stimulation, participants were seated in a chair in a comfortably static position and wore a cap for marking stimulation points. An eight-shaped circular coil connected to a Yiruide CCY-1 stimulator (Yiruide Company Limited, Wuhan, China) was positioned on the hotspot area pressing to the scalp. A single pulse of transcranial magnetic stimulation was delivered to detect the motor-evoked potential (MEP) by electromyographic monitoring from the affected first dorsal interosseous muscle. The minimum stimulation intensity was considered as the resting motor threshold when the collected amplitude was >50 μV in at least 5 out of 10 trials.

After the determination of the resting motor threshold, participants received priming rTMS for the unaffected primary motor cortex. A therapist orientated the handle pointing at a 45° angle to the sagittal line for the CIMT plus active stimulation group or a 90° angle for the CIMT plus sham stimulation group. Parameters for rTMS were as follows: intensity 90% resting motor threshold multiplied by 1 T, frequency 1 Hz for 20 min. For children with absent resting motor thresholds, the fixed resting motor threshold was set as 40% machine output for the consideration of the rough mean resting motor threshold in studies with different groups of people (Delvaux et al., 2003; Ciechanski et al., 2017). That is to say, the stimulation intensity was set as 40% × 90% × 1 T for the participants with absent resting thresholds.

After the active or sham rTMS intervention, a tailor-made restrictive glove was fitted and applied to all the participants on the unaffected hand and forearm (from fingertips to middle forearm) for more than 6 h each day. The restricted hand retained the ability to support or prevent falls (Xu et al., 2012). Motor learning for the affected upper extremity totaled 3 h each day. The participant/therapist ratio of group activities was 3:1 to secure individual guidance. Group activities were age-appropriate and play-based daily living activities to improve children's desire to participate (e.g., tug-of-war, shooting contest, balloon transmission, and desktop cleaning). After the 3-h hospital-centered CIMT training, participants continued family-centered training for 3 h with an exercise program set by therapists to practice with the affected upper extremity under the guidance of caregivers. Telephone follow-up and rehabilitation guidance were conducted every 2 weeks. Daily caregiver-supervised records were followed-up.

### Outcome Assessment

Assessments based on the dimensions of the international classification of functioning, disability, and health (ICF) were performed at the baseline visit, 2 weeks, and 6 months postintervention (Cieza et al., 2019; Angeli et al., 2021). The MEPs and adverse events were assessed to investigate corticospinal excitability changes and safety. Safety was assessed through the self-reporting of symptoms, updating medical records, and physician review.

The manual abilities were classified by the Manual Ability Classification System (for children aged over 4 years old) or Mini-Manual Ability Classification System (for children aged 1–4 years old), the evidence-based standard for upper extremity functional levels (Eliasson et al., 2017; Palisano et al., 2018). The Melbourne Assessment 2 (MA2), a validated tool to evaluate the unaffected upper limb function, was the main outcome measure in this study (Wang et al., 2017). The modified Ashworth scale was performed for the description muscle tone (Meseguer-Henarejos et al., 2018; Zurawski et al., 2019). Bimanual hand performance was assessed by the selective control of the upper extremity scale (Wagner et al., 2016). Perceived changes by caregivers were evaluated by global rating scale and social life ability was evaluated by social life ability scale for Chinese infant–junior school students, which comprised six domains: independent living, athletic abilities, operational abilities, communicative abilities, participation in collective activities, and self-management abilities, with excellent reliability and validity (Zhang et al., 1995). The MEPs in the unaffected motor cortices were measured in the first dorsal interosseous muscles by single-pulse TMS.

Adverse events related to CIMT or rTMS were assessed during the whole study period. A summary of the transient minor adverse events was summarized in prior publications (Gillick et al., 2018).

### Statistical Analysis

The data were analyzed using SPSS version 25.0. For continuous variables, an independent sample *t*-test was performed to compare the baseline data between the two groups which accorded to normal distribution. The ranked variables or variables that did not conform to normal distribution were analyzed by 2 independent samples such as Wilcoxon signed-rank sum test. For categorical variables, the chi-squared test was analyzed. Repeated measures analyses of variance and simple effect analysis were performed for the within-group and between-group differences of upper extremity function, social life ability, perceived changes by parents, and MEP data. Analysis of covariance was used to compute mean differences between the two groups adjusting for baseline. Level information was expressed by frequency and percentage. For every analysis, the significance level was set at *P* < 0.05.

## Results

There were no significant differences in baseline demographic characteristics or functional performance between the two groups (Table 1), with the independent sample *t*-test or Wilcoxon signed-rank sum test (*P* > 0.05).

**Table 1:** Baseline participant characteristics by the group.

	**CIMT + rTMS ^**(+)**^ (*n* = 17)**	**CIMT + rTMS ^**(−)**^ (*n* = 18)**	***P-*value**
Age (m)	50.6 (10.5)	43.83 (12.6)	0.123
Gender, male/female	6/11	8/10	0.594
Left side of hemiparesis, *n* (%)	8 (47.1)	10 (55.6)	0.620
Gross motor function classification system, level I/II	15/2	13/5	0.249
Manual ability classification system, level I/II	11/6	8/10	0.236
The modified Ashworth scale, median (range)	1^+^ (1–3)	2 (1–3)	0.756
**Melbourne assessment 2**			
Range of motion	72.77 (17.37)	68.90 (19.05)	0.535
Accuracy	82.59 (15.16)	74.49 (19.20)	0.177
Dexterity	65.03 (15.34)	59.18 (15.52)	0.271
Fluency	70.31 (13.21)	62.15 (11.85)	0.052
SCUES of affected side	8.71 (2.4)	8.17 (3.6)	0.603
SCUES of the unaffected side	14.00 (2.3)	14.17 (1.7)	0.465
Global rating scale	4.76 (1.8)	4.11 (1.9)	0.304
Standard scores of social life ability scale	10 (1.2)	10 (1.4)	0.930
Magnetic resonance imaging (*n*)	PVL (6), ventricle broadening (3), cyst (1); normal (1), absence (6)	PVL (9); ventricle broadening (4); cyst (2); absence (3)	

*P-value represents between-group differences. Data shown are means (SD) or n (%), unless otherwise stated*.

*CIMT, constraint-induced movement therapy; rTMS, repetitive transcranial magnetic stimulation; SCUES, selective control of upper extremity scale; PVL, periventricular leukomalacia*.

### Improvement of Affected Upper Extremity Function

Most participants had significantly increased MA2 subscale scores (range of motion, accuracy, dexterity, and fluency) at both 2 weeks and 6 months post-intervention compared with the baseline in the two groups (*P* < 0.05, Table 2). The CIMT plus active stimulation group was associated with larger gains in the subscales of accuracy, fluency, and range of motion than the CIMT plus sham stimulation group (*P* < 0.05). Just as important, the difference of average change value of MA2 subscales between groups exceeded the minimum clinically important difference (MCID) of MA2 subscales that has been established (the MCID of MA2 subscales are 2.35, 3.20, 2.09, and 2.22, respectively) (Wang et al., 2017). No significant diffidence was reported between the two groups in the subscale of dexterity (*P* > 0.05).

**Table 2:** Pre- and post-intervention changes in the Melbourne Assessment 2 in the 2 treatment groups.

**Assessments**	**Intervention point**	**CIMT + rTMS ^**(+)**^ (*n* = 17)**	**CIMT + rTMS ^**(−)**^ (*n* = 18)**	***P-*value**
MA2-range of motion	Baseline	72.77 (17.4)	68.90 (19.0)	**0.021**
	2 Weeks	83.44 (13.7)^##^	75.36 (20.5)^#^	
	6 Months	81.25 (14.4)^#^	71.80 (16.1)	
MA2-accuracy	Baseline	82.59 (15.2)	74.44 (19.2)	**0.017**
	2 Weeks	90.35 (10.5)^##^	82.89 (19.1)^##^	
	6 Months	90.59 (10.6)^#^	80.4 (19.3)^#^	
MA2-dexterity	Baseline	65.03 (15.3)	59.18 (15.5)	0.356
	2 weeks	78.28 (14.2)^##^	64.06 (13.8)^#^	
	6 months	73.49 (13.4)^##^	65.25 (13.7)^##^	
MA2-fluency	Baseline	70.31 (13.2)	62.15 (11.8)	**0.020**
	2 Weeks	79.83 (11.1)^##^	74.60 (11.4)^##^	
	6 Months	79.27 (12.8)^##^	68.26 (11.0)^##^	
SCUES (affected)	Baseline	8.71 (2.4)	8.17 (3.6)	0.742
	2 Weeks	12.59 (2.5)^##^	11.28 (3.5)^##^	
	6 Months	10.76 (2.2)^##^	9.56 (2.5)	
SCUES (unaffected)	Baseline	14.00 (2.3)	14.17 (1.7)	0.451
	2 Weeks	13.24 (2.4)	12.94 (3.6)	
	6 Months	14.06 (1.5)	13.89 (1.2)	

*P-value represents between-group differences (the bold values represent P <0.05). Values are reported as mean (SD). Within-group and between-group differences were analyzed with repeated measures analyses of variance*.

# *Significantly different than baseline, P <0.05*.

## *Significantly different than baseline, P <0.01*.

*CIMT, constraint-induced movement therapy; rTMS, repetitive transcranial magnetic stimulation; MA2, the modified Melbourne assessment 2; SCUES, selective control of upper extremity scale*.

**Figure 2:**
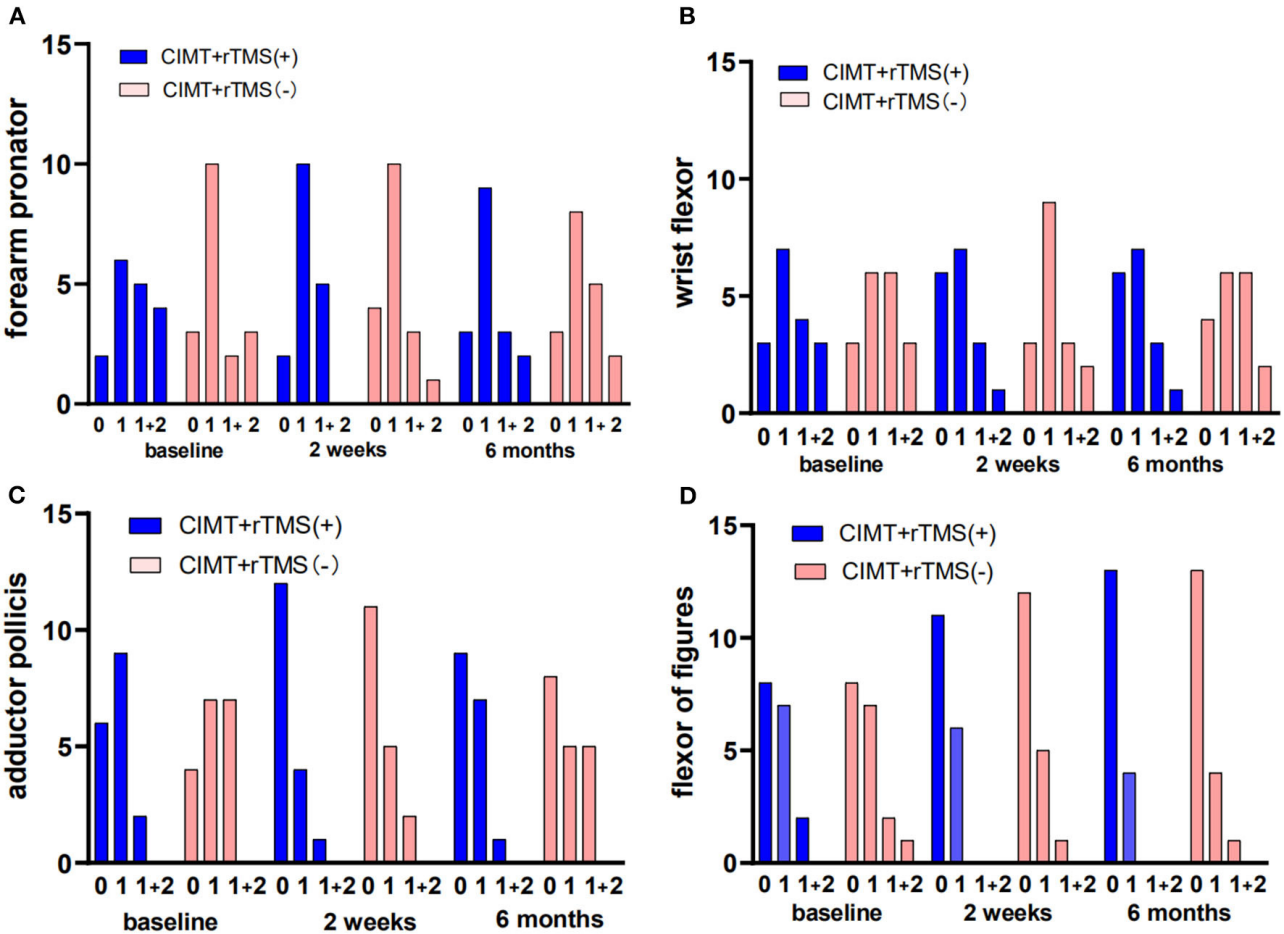
Changes of muscle tone of the affected upper extremity in the constraint-induced movement therapy (CIMT) plus active stimulation group and sham stimulation group. **(A)** Muscle tone of forearm. **(B)** Muscle tone of wrist. **(C)** Muscle tone of thumb. **(D)** Muscle tone of the other fingers. rTMS, repetitive transcranial magnetic stimulation.

For muscular tone, no treatment-related change emerged in the modified Ashworth scale (forearm, wrist, thumb, and fingers) in the two groups (*P* > 0.05, Figure 2).

### Bimanual Performance

Although most participants had increased selective control of the affected upper extremity scale scores, there was no significant difference between the two groups (*P* > 0.05, Table 2). As for the unaffected upper extremity, there was no significant within-group and between-groups difference (*P* > 0.05).

### Social Life Ability and Perceived Changes by Caregivers

**Figure 3:**
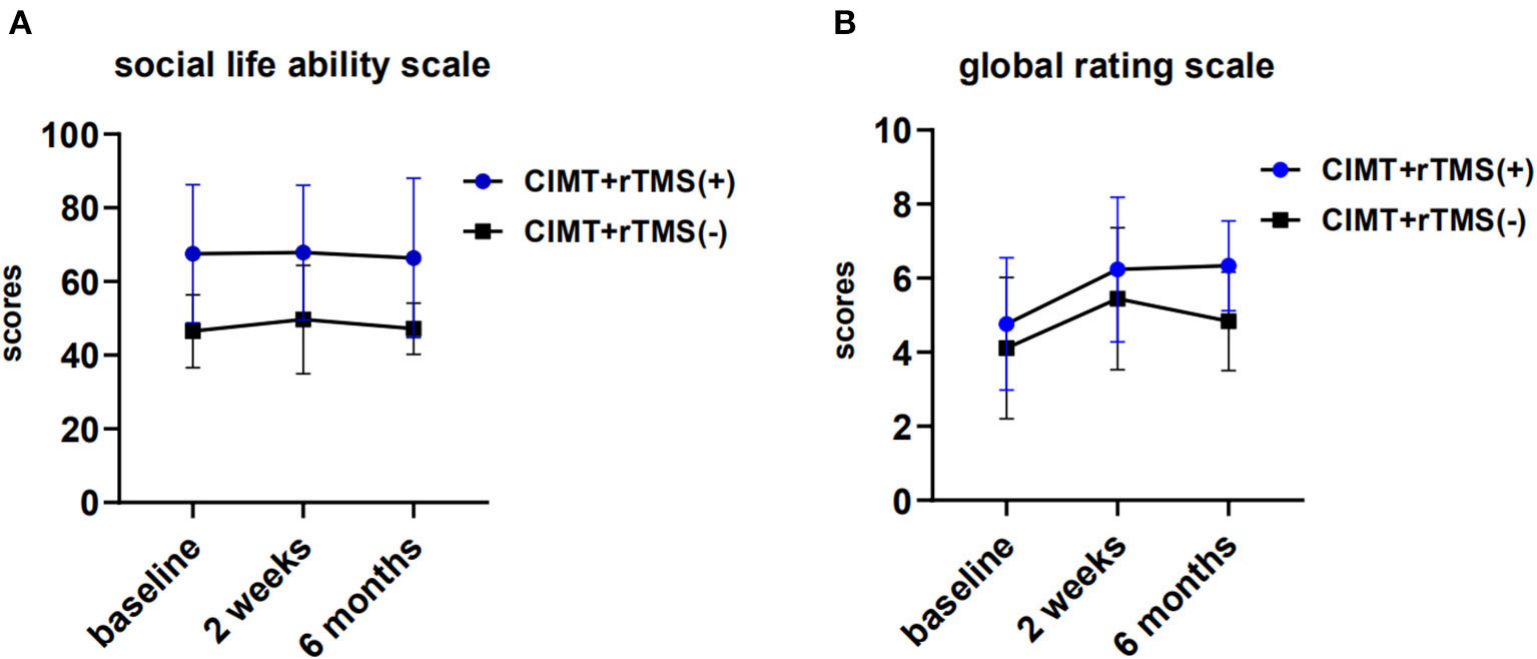
Perceived changes by caregivers and changes of activity of daily living in the constraint induced movement therapy (CIMT) plus active stimulation group and sham stimulation group. **(A)** Changes of global rating scale. **(B)** Changes of social life ability scale for Chinese infant-junior school student. rTMS, repetitive transcranial magnetic stimulation.

For the social life ability scale, there were no significant within-group and between-group differences between the two groups (*P* > 0.05; Figure 3A). We found that the global rating scale scores achieved clinically significant gains at 2 weeks of post-intervention in both the groups (*P* < 0.01), even though there was no significant between-group difference (*P* > 0.05; Figure 3B).

### Motor-Evoked Potential Outcomes

To investigate the correlations between MEP outcomes and hand function after the intervention of CIMT combined with active rTMS stimulation, we compared MA2 outcomes between children with (*n* = 7) and without (*n* = 10) MEPs in the lesioned hemisphere at 2 weeks of post-intervention. No significant difference emerged between the groups (*P* > 0.05, Table 3).

**Table 3:** Comparison of upper extremity function between the groups with or without MEPs at 2 weeks of postintervention.

**Measurements**	**Intervention point**	**With MEP (*n* = 7)**	**Without MEP (*n* = 10)**	**Between-group comparison (*P*)**
MA2-range of motion	Baseline	80.42 (4.90)	78.27 (8.51)	0.826
	2 Weeks	87.83 (4.41)	82.54 (6.40)	
MA2-accuracy	Baseline	76.57 (18.82)	86.86 (13.41)	0.426
	2 Weeks	88.57 (13.35)	90.29 (9.48)	
MA2-dexterity	Baseline	58.64 (18.59)	74.47(8.24)	0.381
	2 Weeks	78.59 (19.39)	82.57 (8.42)	
MA2-fluency	Baseline	69.38 (17.14)	75.51 (9.29)	0.329
	2 Weeks	74.53 (19.48)	80.03 (6.40)	
SCUES (affected)	Baseline	7.71 (2.36)	10.00 (1.91)	0.648
	2 Weeks	12.71 (2.87)	13.43 (2.15)	
SCUES (unaffected)	Baseline	14.29 (1.89)	15.00 (0.00)	0.288
	2 Weeks	12.71 (2.87)	13.86 (2.27)	

*Values are reported as mean (SD)*.

*MEP, motor-evoked potential; CIMT, constraint-induced movement therapy; rTMS, repetitive transcranial magnetic stimulation*.

### Safety

Headache occurred in one participant, which was relieved after several minutes. No participants reported severe adverse events such as epileptic seizures or behavioral problems during the study session.

## Discussion

We examined the effect of the intervention of CIMT combined with rTMS on preschool children with UCP and found that the addition of rTMS exaggerated the effect on the affected upper extremity function induced by CIMT. No serious adverse events occurred during the study period, only one participant reported a self-limiting headache.

In this study, most participants experienced improvements in the affected upper extremity function after 2 weeks and 6 months post-intervention. Greater improvement in accuracy, fluency, and range of motion in the CIMT plus active stimulation group, suggested a greater impact of CIMT combined with rTMS than CIMT alone, which is consistent with the previous study in school-age children (Kirton et al., 2016). Young children with UCP are often affected by joint reaction and mirror movements (Ismail et al., 2017). Hence, it is still necessary to carry out effect-oriented trials of CIMT combined with rTMS in younger children, and our results complemented this evidence in preschool children with UCP. Even though no significant difference in dexterity and selective control of the upper extremity scale of the affected upper extremity were reported between the two groups, participants who received CIMT combined with active stimulation had more favorable mean scores 2 weeks postintervention. Notably, improvements measured with MA2 sustained for 6 months in this study may reflect long-term depression of 1-Hz rTMS in corticospinal excitability. The maintained after-effect, which may be relevant to a complex scenario (e.g., gene activation/regulation, *de-novo* protein expression, and postsynaptic excitability state), is the rationale for rTMS applications as a clinical tool (Cirillo et al., 2017; Baur et al., 2020).

The muscle tone was not reported with significant differences between groups. The previous study has indicated the positive effect of 10-Hz rTMS on muscle tone of children with cerebral palsy (Rajak et al., 2019). In light of the proven safety of low-frequency rTMS, we adhered to established principles of 1-Hz rTMS applied to the unaffected motor cortex (Emara et al., 2010; Gillick et al., 2014a). Rossi et al. had compared the safety between high-frequency and low-frequency rTMS and found that induction of seizures was with 1.4% and crude risk estimate in epileptic patients and <1% under high-frequency stimulation in patients without the history of seizures, yet was hardly reported in studies with low-frequency stimulation (Gillick et al., 2014a). In line with the evidence of low-frequency rTMS, no serious adverse event was reported in this study. For developing brains, safety deserves to be handled with the utmost seriousness, and more studies of low-frequency rTMS on this group are warranted.

A previous study reported improvements in quality-of-life measures in children older than six (Gillick et al., 2014b; Kirton et al., 2016; Rich et al., 2016). However, we did not find any significant differences in social life ability scale scores and perceived changes by caregivers between the two groups. One of the potential factors to consider was the educational environment in China. Many Chinese caregivers, especially grandparents, usually overprotect their kids and are used to reducing the opportunities of their children to complete the tasks in life by themselves, which may limit the improvements to the children's social abilities to a certain extent. What is more, the optimal timing of follow-up for clinically relevant change of CIMT combined with rTMS is not well understood in young children. A longer follow-up period and more follow-up time points may be important for the understanding of clinically relevant change.

It was shown that MEPs were detected only from some participants. We wondered if children with absent MEP on the affected side do worse than the others after the intervention of CIMT combined with active rTMS. Interestingly, we did not find significant differences in upper extremity function between the groups with or without MEP, which provides a train of thought to search for an optimal fixed motor threshold for young children with absent MEP. On the other hand, the reason for MEP absence in young children is not well understood yet. We presumed that the high level of motor cortex excitability and the difficulty for young children to maintain relaxed muscles may be important resources. An increased understanding of the developmental neurophysiological processes in preschool children with cerebral palsy is essential for the establishment of neuromodulation principles. Considering the difficulty of measuring the MEPs for preschool children, our study may be a beneficial exploration of the rTMS parameters for this group.

In addition, studies reported that the integrity of underlying brain anatomy and various brain disorders could potentially influence the distribution of current across the scalp, which may contribute to the variable efficacy of rTMS in children with brain disorders (Rossi et al., 2009; Klomjai et al., 2015). Importantly, a large number of studies have focused on UCP attributed to perinatal stroke, although complicated factors may play an important role in cerebral palsy (e.g., leukomalacia and intracranial hemorrhage in infants). Participants in the study were represented with various brain disorders, expanding the chance of variable efficacy of rTMS. Furthermore, consistent with the adult stroke model, current models considered interhemispheric balance in young children as a spectrum, rather than a dichotomy. Pino et al. (2014) demonstrated that the cerebral structural reserve (preservation of neural pathways and connections) was important to cerebral plasticity. The chance is that the treatment effect is related to interhemispheric balance rather than the simple interhemispheric competition model. In this context, the determination of brain damage is important to the rTMS effect.

Limitations of this study embodied the modest sample size, the insufficient follow-up time points, and lack of subgroups for lesion location of brain and age. Still, there was no formal assessment of potential complications and the impact of parental education and social background on treatment. Different requirements and expectations of the parent may lead to bias in some subjective indicators.

Concerns about the deeper influence of age and lesion location of the brain on CIMT combined with rTMS warrant further investigation in studies. With the combination of neuroimaging techniques, we can observe the changes of cerebral blood flow and molecular biology in the course of rTMS action, thus providing more help for studying the mechanism of rTMS and the best treatment parameters.

## Conclusion

The rTMS combined with CIMT has a superimposed therapeutic effect on the affected hand function in preschool children with UCP attributed to various brain disorders, which is safe and worthy of promotion among this group of children.

## Data Availability Statement

The raw data supporting the conclusions of this article will be made available by the authors, without undue reservation.

## Ethics Statement

The studies involving human participants were reviewed and approved by Guangzhou Women and Children's Medical Center Research Ethics Committee (Approved No. 14300). Written informed consent to participate in this study was provided by the participants' legal guardian/next of kin.

## Author Contributions

KX conceived this study, contributed to the study design, and attributed to project management and fund procurement. QW and TP wrote this manuscript and performed data collection. LL and YX generated the figures and tables. PZ contributed to guidance on English writing. XY and YZ performed data analysis. CF, SH, YH, and HZ carried out the literature search. YL, HT, and LH contributed to participant recruitment. KX and LH revised the manuscript. All authors have read and approved the content of the manuscript.

## Funding

This study was supported by the National Natural Science Foundation of China (81902309 and 81672253), the Natural Science Foundation of Guangdong Province (2021A1515011303, 2021A1515012543, and 2019A1515010420), the Basic and Applied Basic Foundation of Guangdong Province (2020A1515110435), funds from the Guangzhou Municipal Science and Technology Project (202102010205 and 202102020581), and the Featured Clinical Technique of Guangzhou (2019TS55). The funders played no role in the design, conduct, or reporting of this study.

## Conflict of Interest

The authors declare that the research was conducted in the absence of any commercial or financial relationships that could be construed as a potential conflict of interest.

## Publisher's Note

All claims expressed in this article are solely those of the authors and do not necessarily represent those of their affiliated organizations, or those of the publisher, the editors and the reviewers. Any product that may be evaluated in this article, or claim that may be made by its manufacturer, is not guaranteed or endorsed by the publisher.
